# Mathematical Models of Cancer: When to Predict Novel Therapies, and When Not to

**DOI:** 10.1007/s11538-019-00640-x

**Published:** 2019-07-23

**Authors:** Renee Brady, Heiko Enderling

**Affiliations:** Department of Integrated Mathematical Oncology, H. Lee Moffitt Cancer Center and Research Institute, Tampa, FL 33647 USA

**Keywords:** Mathematical oncology, Treatment prediction, Optimization, Parameter estimation

## Abstract

The number of publications on mathematical modeling of cancer is growing at an exponential rate, according to PubMed records, provided by the US National Library of Medicine and the National Institutes of Health. Seminal papers have initiated and promoted mathematical modeling of cancer and have helped define the field of mathematical oncology (Norton and Simon in J Natl Cancer Inst 58:1735–1741, 1977; Norton in Can Res 48:7067–7071, 1988; Hahnfeldt et al. in Can Res 59:4770–4775, 1999; Anderson et al. in Comput Math Methods Med 2:129–154, 2000. 10.1080/10273660008833042; Michor et al. in Nature 435:1267–1270, 2005. 10.1038/nature03669; Anderson et al. in Cell 127:905–915, 2006. 10.1016/j.cell.2006.09.042; Benzekry et al. in PLoS Comput Biol 10:e1003800, 2014. 10.1371/journal.pcbi.1003800). Following the introduction of undergraduate and graduate programs in mathematical biology, we have begun to see curricula developing with specific and exclusive focus on mathematical oncology. In 2018, 218 articles on mathematical modeling of cancer were published in various journals, including not only traditional modeling journals like the Bulletin of Mathematical Biology and the Journal of Theoretical Biology, but also publications in renowned science, biology, and cancer journals with tremendous impact in the cancer field (Cell, Cancer Research, Clinical Cancer Research, Cancer Discovery, Scientific Reports, PNAS, PLoS Biology, Nature Communications, eLife, etc). This shows the breadth of cancer models that are being developed for multiple purposes. While some models are phenomenological in nature following a bottom-up approach, other models are more top-down data-driven. Here, we discuss the emerging trend in mathematical oncology publications to predict novel, optimal, sometimes even patient-specific treatments, and propose a convention when to use a model to predict novel treatments and, probably more importantly, when not to.

## Introduction

### The Past and Present of Mathematical Oncology

**Fig. 1:**
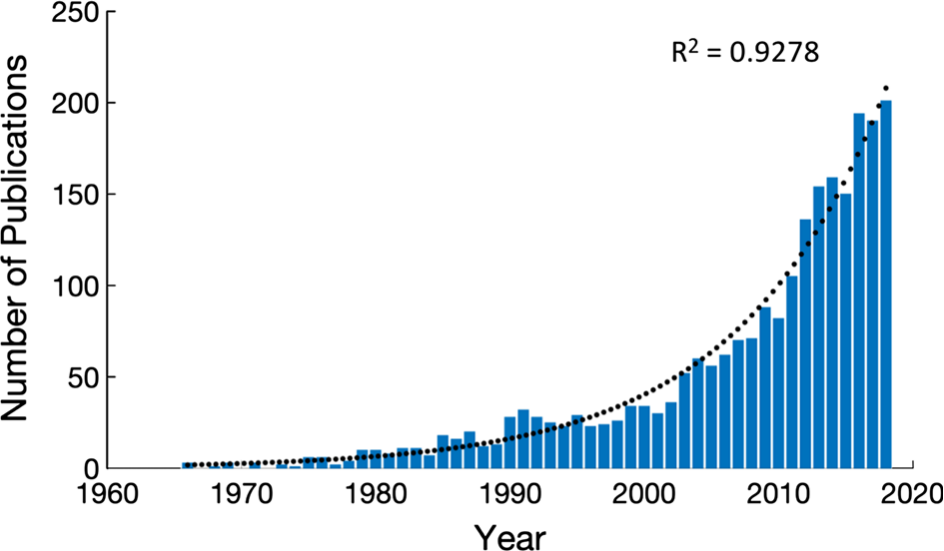
PubMed query for “Mathematical model” AND (“cancer” OR “tumor”), accessed 3/1/19. The trend line indicates an exponential increase in number of mathematical oncology publications since 1968 (Color figure online)

Mathematical modeling in cancer has a long history as reviewed in multiple publications (Araujo and McElwain 2004; Lowengrub et al. 2010; Altrock et al. 2015; Friedman 2004). According to PubMed records provided by the US National Library of Medicine and the National Institutes of Health, the number of publications on mathematical modeling of cancer is growing at an exponential rate (Fig. 1). Early conceptual mathematical models highlighted the universal dynamics of cancer growth (Agur et al. 2002; Brú et al. 2003; Guiot et al. 2003) and the emergent properties of proliferation and invasion mechanisms (Anderson et al. 2006; Hillen 2006). As cancer is an umbrella term for more than 100 different diseases with different intrinsic dynamics and unique environmental and ecological niches, mathematical models have begun to focus on the properties of specific cancers such as leukemia (Michor et al. 2005) and glioma (Eikenberry et al. 2010) or cancers of the breast (Enderling et al. 2006), prostate (Swanson et al. 2001), or bladder (Bunimovich-Mendrazitsky et al. 2008) among many others. To this success, model parameters need to be carefully assigned, often from the experimental and the clinical literature of the specific cancer. Numerous mathematical oncology publications stand out by virtue of their truly integrative nature and deliberate and interdisciplinary model calibration and validation effort (Kozusko et al. 2001; Anderson et al. 2009; Leder et al. 2013; Marusyk et al. 2014; Prokopiou et al. 2015; Poleszczuk and Enderling 2018; Kaznatcheev et al. 2019). One of the mainstays of mathematical oncology is modeling of the various oncological treatments including surgery (Hanin et al. 2015; Enderling et al. 2005), radiation therapy (McAneney and O’Rourke 2007; Kempf et al. 2010; Alfonso et al. 2014; Gao et al. 2013), chemotherapy (Powathil et al. 2007; Castorina et al. 2009; Hinow et al. 2009; Vainstein et al. 2011; Powathil et al. 2012), anti-angiogenic therapy (Poleszczuk et al. 2015; Sachs et al. 2001; Poleszczuk et al. 2011; Hutchinson et al. 2011), virotherapy (Dingli et al. 2006; Friedman et al. 2006; Mahasa et al. 2017; Santiago et al. 2017), and immunotherapy (Kogan et al. 2012; Elishmereni et al. 2011; Nani and Freedman 2000; Kuznetsov and Knott 2001; Castiglione and Piccoli 2007; Kirschner and Tsygvintsev 2009) as well as their numerous possible combinations (Hawkins-Daarud et al. 2015; Bunimovich-Mendrazitsky et al. 2011; Alfonso et al. 2019).

Several agencies have provided funding mechanisms for mathematical oncology, including the National Science Foundation (NSF) and the National Cancer Institute (NCI) in the USA, the Engineering and Physical Sciences Research Council (EPSRC) and Cancer Research UK, the German Cancer Research Center (DKFZ), and multinational frameworks within the European Union. Several mathematical modeling research groups and even mathematical oncology departments have become established in cancer centers and medical schools around the world. As a result, we begin to witness the translation and evaluation of mathematical model-derived treatment protocols in prospective clinical trials (NCT03557372, NCT03768856, NCT03656133) (Leder et al. 2013; Prokopiou et al. 2015; Alfonso et al. 2018). Mathematical exploration of the evolutionary dynamics underlying the development of prostate cancer resistance to hormone therapy (Cunningham et al. 2011; Gatenby et al. 2009; Gatenby and Brown 2018) led to a clinical trial of adaptive hormone therapy with treatment holidays to prevent competitive release of resistant cancer cells (NCT02415621). Early results indicate that most patients maintained stable oscillations of tumor burdens, thereby significantly increasing time to progression from 13 to at least 27 months. Interestingly, on average, patients received less than half of the treatment dose than conventional continuous therapy (Zhang et al. 2017). With these mathematical oncology success stories, cancer biologists and oncologists begin to embrace mathematical modeling as a valuable methodology. With this, the mathematical oncology community has the opportunity and responsibility to clearly identify the purpose of developed models and critically evaluate and discuss if model predictions are an academic exercise or have true translational merit.

As many mathematical models have become increasingly complex through the iterative inclusion of the growing biological knowledge, the field of mathematical modeling has slipped into parameterization from multiple sources often mixing cancer types, experimental conditions, and even spatio-temporal scales. It has become routine to provide references for parameter values from other theoretical studies; in selected instances, the parameter value reference trail may follow back many publications to an initially “assumed” value without any biological or clinical data supporting the assumption. Incorrectly calibrated and unvalidated mathematical models risk simulating cancer growth and treatment response dynamics that are, albeit being interesting, biologically and clinically unrealistic. Yet, it has become an increasing trend to use inadequately parameterized mathematical cancer models to predict untested treatment protocols, to propose optimize therapy combinations and, in some cases, to develop personalized optimal therapy approaches.

*Bottom*-*up* phenomenological mathematical models often formalize biological mechanisms. Model analysis and numerical simulations demonstrate evolving population level dynamics based on these mechanisms. This allows the study of complex biological and mathematical systems, and how perturbations to individual mechanisms or rate constants qualitatively change tumor growth or treatment response. Vis-à-vis the *bottom*-*up* approach is the *top*-*down* approach, where population level dynamics are used to infer the mechanisms that most likely underlie the observed data. With sparse data, this often limits complexity of mathematical oncology models. Another crucial distinction to make is mathematical oncology vs. oncological mathematics. While the former uses mathematical modeling as a purposely built tool to help answer specific oncological questions, the latter uses cancer biology to motivate development of interesting mathematics. Both approaches provide valuable scientific contributions and are not necessarily mutually exclusive. However, as many cancer modelers lack access to high-resolution cancer biology or oncology data including independent training and validation data sets, many models are merely academic and not positioned to speculate on optimal therapy.

### The Future of Mathematical Oncology Predictions for Novel Cancer Therapies

**Fig. 2:**
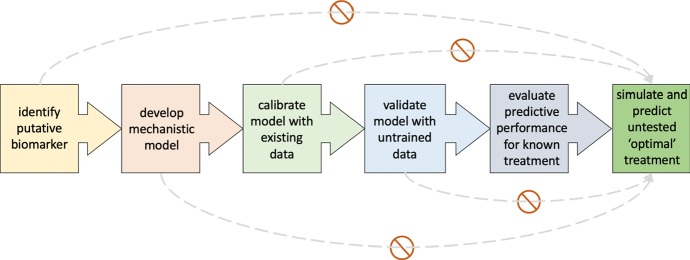
Proposed pipeline for predicting novel, potentially optimal therapy. Dashed arrows mark commonly used shortcuts to predictions that should be avoided in translational models to predict novel treatments (Color figure online)

Before a mathematical model can make reliable, testable and translational predictions about novel therapeutic doses, treatment protocols or combination therapies, we propose that six successive steps have to be followed (Fig. 2):

1. Identify a putative biomarker

To simulate cancer and cancer therapy, a biomarker of tumor burden needs to be identified. This may be the number of cancer cells in a petri dish or in the patient’s blood for liquid tumors, tumor volume derived from medical images or caliper measurements from mouse experiments for solid cancers, or surrogate markers such as prostate-specific antigen (PSA) in liquid biopsies. In contrast to statistical models that correlate random variables (such as pre-treatment tumor size with treatment outcome), mathematical modeling simulates the dynamics of the tumor and their underlying mechanisms. Therefore, the data of putative biomarkers for mechanistic modeling should be temporally resolved.

2. Develop mechanistic model

The change of the putative biomarker over time is described using dynamic models such as mechanistic differential equations or cellular automata or agent-based models (here, we focus on differential equations but the application to discrete models is intuitive). If only temporal data are available, ordinary differential equations (ODE) are often sufficient. Partial differential equations (PDE) should only be used if spatio-temporally resolved data are available, or temporal dynamics alone are insufficient to explain the observed biomarker dynamics. The number of model variables and parameters should be determined with utmost care and limited based on the available data. Information criteria offer invaluable analyses to balance model complexity with degrees of freedom (Akaike 1974; Ludden et al. 1994).

If longitudinal data are limited (as often in clinical studies), non-mechanistic or statistical models may be considered that simulate the dynamics of the biomarker without resolution of the underlying mechanisms. Such models, however, will have limited ability to predict novel treatment protocols beyond considerations for treatment holidays.

3. Calibrate model with existing data

The mathematical model is only suitable to simulate and predict novel treatment protocols if it can fit and predict the data of known therapies. If possible, model parameters should not be taken from the empirical wisdom or the literature but derived from the data within realistic bounds. A variety of machine learning and established statistical methods are already available to identify model parameters for simple models. To further aid in parameter estimation for larger models and across multiple data sets, however, major advances are still required to reliably calibrate mathematical models. Model analysis should be performed to test parameter identifiability (Eisenberg and Jain 2017). Non-identifiable parameters or parameters with low sensitivity may be represented as (non-)linear combination of other model parameters or assigned nominal values from the appropriate literature.

For individual patient data and models built with the intent to predict patient-specific treatment protocols, it is important to identify which parameters drive individual response dynamics, and which parameters can be held uniform across a patient population. As above, the number of patient-specific parameters should be kept to a minimum to be able to learn these parameter values from patient data to simulate and predict personalized treatment protocols.

4. Validate model with untrained data

Once calibrated with data, the mathematical model must be validated against independent data. The learned model parameters should be held constant, and the model’s ability to fit untrained data should be evaluated via methods such as R^2^ analyses. If independent training and testing data sets are not available, data should be randomly stratified into model calibration and validation cohorts. For smaller data sets, leave-one-out studies may be required to learn and test model parameters. This step provides an internal control and prevents overfitting to training data.

5. Evaluate predictive performance for known treatment

A model’s ability to fit data does not imply that it is predictive. Many models may fit data equally well but make different predictions forward in time (Murphy et al. 2016). Before a mathematical model should be used to predict novel treatments, the model must be able to predict responses to treatment protocols for which data are available. As above, an independent prediction evaluation cohort is the gold standard, but random stratification of a single data set into model training, validation, and prediction performance evaluation may be required and acceptable. How to evaluate model prediction performance depends on the purpose of the model. If the model aims to predict binary events (response, resistance, survival), statistical tools such as the concordance index or area under the receiver operating characteristic curve may be applicable (Steyerberg et al. 2010). To further understand and assess clinical utility, the positive predictive value (PPV) and negative predictive value (NPV) for model prediction should be calculated (Janes et al. 2015). If and only if the predictive performance for known treatment responses and outcomes is sufficiently high (a conventional notion of acceptable cutoffs for predictive performance is yet to be established in the field of mathematical oncology), models may be used to simulate and predict untested treatments.

6. Simulate and predict untested treatments

To use calibrated and validated model parameters to simulate alternative treatments, it is important to limit the exploration space to treatments that can be derived from the calibrated and validated model. Treatments that the model was not trained to predict should not be simulated. Furthermore, if, for example, the training data contain only single-dose levels and the biological dose response curves are unknown, predictions into untested doses should be met with caution and limited confidence.

While it is straightforward to simulate arbitrary treatment protocols, clinical feasibility (for example, radiation therapies can rarely be delivered more than twice a day or on the weekend due to logistical constraints) and biologically important bounds (such as drug agent half-life or toxicity) must be honored. Additionally, when comparing predicted responses to innovative treatments with the data, one can only draw conclusions about the evaluated regimes. To claim ‘optimal therapy,’ rigorous optimal control approaches and an exhaustive analysis should be provided.

## Conclusions

Mathematical oncology has contributed significant scientific insights into the dynamics of tumor growth and treatment response and is uniquely positioned to help map the multidimensional treatment response space (Enderling et al. 2018). The art of the mathematical oncology profession must deploy a standard to only predict novel treatments if the model is properly trained and validated to do so. We call for the convention to up-front identify model purpose and model predictions as either ‘academic’ or ‘translational’ in nature. Shortcuts to translational treatment predictions (Fig. 2) may hurt the field of mathematical oncology that is in the advent of earning the trust of oncologists and, ultimately, may hurt patients that would be prospectively treated with ill-informed, model-suggested treatment protocols.
